# Health Belief Model Perspective on the Control of COVID-19 Vaccine Hesitancy and the Promotion of Vaccination in China: Web-Based Cross-sectional Study

**DOI:** 10.2196/29329

**Published:** 2021-09-06

**Authors:** Hao Chen, Xiaomei Li, Junling Gao, Xiaoxi Liu, Yimeng Mao, Ruru Wang, Pinpin Zheng, Qianyi Xiao, Yingnan Jia, Hua Fu, Junming Dai

**Affiliations:** 1 Department of Preventive Medicine and Health Education School of Public Health Fudan University Shanghai China

**Keywords:** COVID-19 pandemic, vaccination behavior, vaccine hesitancy, health belief model

## Abstract

**Background:**

The control of vaccine hesitancy and the promotion of vaccination are key protective measures against COVID-19.

**Objective:**

This study assesses the prevalence of vaccine hesitancy and the vaccination rate and examines the association between factors of the health belief model (HBM) and vaccination.

**Methods:**

A convenience sample of 2531 valid participants from 31 provinces and autonomous regions of mainland China were enrolled in this online survey study from January 1 to 24, 2021. Multivariable logistic regression was used to identify the associations of the vaccination rate and HBM factors with the prevalence of vaccine hesitancy after other covariates were controlled.

**Results:**

The prevalence of vaccine hesitancy was 44.3% (95% CI 42.3%-46.2%), and the vaccination rate was 10.4% (9.2%-11.6%). The factors that directly promoted vaccination behavior were a lack of vaccine hesitancy (odds ratio [OR] 7.75, 95% CI 5.03-11.93), agreement with recommendations from friends or family for vaccination (OR 3.11, 95% CI 1.75-5.52), and absence of perceived barriers to COVID-19 vaccination (OR 0.51, 95% CI 0.35-0.75). The factors that were directly associated with a higher vaccine hesitancy rate were a high level of perceived barriers (OR 1.63, 95% CI 1.36-1.95) and perceived benefits (OR 0.51, 95% CI 0.32-0.79). A mediating effect of self-efficacy, influenced by perceived barriers (standardized structure coefficient [SSC]=−0.71, *P*<.001), perceived benefits (SSC=0.58, *P*<.001), agreement with recommendations from authorities (SSC=0.27, *P*<.001), and agreement with recommendations from friends or family (SSC=0.31, *P*<.001), was negatively associated with vaccination (SSC=−0.45, *P*<.001) via vaccine hesitancy (SSC=−0.32, *P*<.001).

**Conclusions:**

It may be possible to increase the vaccination rate by reducing vaccine hesitancy and perceived barriers to vaccination and by encouraging volunteers to advocate for vaccination to their friends and family members. It is also important to reduce vaccine hesitancy by enhancing self-efficacy for vaccination, due to its crucial mediating function.

## Introduction

COVID-19 has spread worldwide, causing more than 88 million infections and more than 1.9 million deaths as of January 2021 [1]. Due to the lack of effective treatments, the development and use of a new COVID-19 vaccine has become an important strategy to control the epidemic. Since COVID-19 broke out, according to the World Health Organization (WHO), 60 new coronavirus-inactivated vaccines and more than 10 nucleic acid vaccines, vector vaccines, and protein subunit vaccines have been developed [2]. Vaccination is recognized as the most successful and cost-effective public health intervention in the world today, and it has made a very large contribution to improving global health by reducing the incidence and deaths of many infectious diseases [3,4]. China and the whole world are experiencing the third wave of epidemics, so it is especially important to establish herd immunity by vaccinating against COVID-19 [5].

On December 30, 2020, the first homegrown COVID-19 vaccine in China was approved for marketing by the China National Medical Products Administration, and open volunteer vaccination to the public was announced through official media. On January 9, 2021, the National Health Commission promised free vaccinations for the Chinese population [6]. As of February 2021, the COVID-19 vaccine in China is suitable for people aged 18 to 59 years; the COVID-19 vaccine is not suitable for pregnant women, lactating women, and people with the following conditions: acute stages of fever, infections and other diseases, immune deficiency or immune disorders, serious liver and kidney diseases, hypertension, diabetic complications, and malignant tumors with uncontrolled drugs [7]. As of February 2021, common adverse reactions to vaccines in China mainly include headache, fever, local redness or lumps at the inoculation site, and cough, as well as loss of appetite, vomiting, and diarrhea in some people [8]. In first month of COVID-19 vaccinations, up to January 26, 2021, 22.8 million doses of the COVID-19 vaccine were administered in China, and less than 5% of the vaccine-eligible population among them, the main group, was at high risk of infection in all regions [9]. Considering the occupational exposure risk of COVID-19 infection, some populations with priority for vaccination were those with occupations at border ports, in key places such as international and domestic transportation, and in key industries such as medical and health care as well as basic social operation services. These populations are mass vaccinated on the basis of individual willingness [6]. According to the director of the National Health Commission, National Bureau of Disease Control and Prevention, all residents could be vaccinated in an orderly manner where there is an ample supply of vaccine and where vaccination units are health service centers, township health centers, or general hospitals located in their respective jurisdictions. Local governments have been required to make public in a timely manner the vaccination sites and units that can administer vaccines in their respective jurisdictions, including their locations and service hours [8]. From the beginning of 2020 to February 2021, the State Council Information Office has held regular press conferences to invite experts from relevant departments to brief the population on the joint prevention and control of COVID-19 [10]. Not only have the daily numbers of new cases, close contacts, patients who recovered, and patients who died been announced, but the latest number of vaccinated people as well as side effects and psychological changes following vaccination have also been announced. In addition to communications at the national level, provinces, municipalities, and autonomous regions released the latest information about the epidemic through various channels, such as press conferences or short videos, specific to their own situations, to ensure mastery and understanding of information regarding the epidemic and vaccines [11,12]. As of May 2021, 6 months after countries had begun carrying out vaccinations, the global number of COVID-19 vaccinations has exceeded 1.5 billion doses. Among them, nearly 60% are concentrated in China (420 million doses), the United States (270 million doses), and India (180 million doses). Except for a few countries with a vaccination rate exceeding 50% (eg, Israel), most countries in the world have a vaccination rate below 20% [13]. According to a recent study, predicted vaccine coverage of 55% to 82% of the population is needed to achieve COVID-19 herd immunity [5]. In addition to the supply of vaccines, individuals’ psychological mechanisms of vaccine behavior are particularly critical to vaccination [14]. Therefore, it is of great significance to explore the possible influencing factors of individuals’ vaccination willingness when vaccination rates are low in order to improve COVID-19 vaccination willingness and coverage in China and other parts of the world.

Although vaccines are currently an effective means of improving global health, in many parts of the world there are still quite a few people who question the necessity of vaccination, postpone vaccination, or even refuse vaccination; this is especially true when vaccines first came to market and were met with considerable hesitation and even outright opposition [15]. In 2012, the WHO established the Strategic Advisory Group of Experts (SAGE) working group to address and define vaccine hesitancy and its scope [16]. Vaccination hesitancy was defined as the refusal or delay of vaccination when vaccination services were available [17], and vaccination hesitancy was listed among the 10 threats to global health in 2019 [18]. Vaccine hesitancy is reflected in many factors, including confidence in the efficacy and safety of the vaccine and in the health service system providing the vaccine, such as the reliability and competence of the health service system and the professionals involved in the vaccination service [19]. In the first month after vaccines became available to all vaccine-eligible members of the Chinese population, a nationwide cross-sectional study reported the prevalence of COVID-19 vaccination hesitancy to be 35.5%. After an instance of illegal marketing of vaccines, 32.4% of parents became hesitant of vaccines [20]; rapid sociocultural changes have also contributed to vaccine hesitancy [21,22]. A study on COVID-19 vaccine hesitancy of Italian college students showed that among the 735 students who answered questions about their vaccination intentions, more than 1 in 10 students showed hesitancy [23]. An investigation during Israel’s mandatory quarantine revealed that nurses and medical workers showed high levels of vaccine hesitancy [24]. According to a literature review, 68.4% of the global population is willing to receive the vaccination [25].

Recent studies of factors associated with COVID-19 vaccination have identified a number of demographic, cognitive, and psychosocial factors, including age, gender, educational level, insurance status, attitudes toward the vaccine, confidence in government information, perceived susceptibility to COVID-19, and perceived benefits and side effects of the vaccine [26,27]. In the current age of Web 2.0, the spread of false news about vaccine safety and validity on social media, such as that COVID-19 vaccination can affect individuals’ reproductive function, influence vaccination willingness and confidence [28]. Several typical behavioral theories, such as the health belief model (HBM), the theory of planned behavior (TPB) [29], and the diffusion of innovation theory (DIT) [14], have been used to explain COVID-19 vaccination intent combined with demographic, cognitive, and psychosocial factors. The HBM is a widely used theory that proposes a variety of psychological factors that affect people's health protective behaviors, such as attitudes, beliefs, and intentions [30-32]. The HBM assumes that health-related actions depend on the simultaneous occurrence of three factors [33]: (1) the presence of sufficient motivation (or health concern) to make the health problem salient or relevant, (2) the belief that a person is vulnerable to serious health problems or the sequelae of that illness or condition is often referred to as a perceived threat, and (3) believing that following a specific health recommendation will help reduce the perceived threat at a subjectively acceptable cost. The TPB assumes that an individual's behavioral posture, activity attraction, and behavioral control jointly affect and direct the individual's behavior [34]. The DIT aims to disseminate innovation awareness, technology, or innovative ideas related to the masses, so that patients can develop innovative thinking or health awareness. In recent years, the DIT has been gradually introduced into medical and health industries, mainly for the guidance of health education strategies [14]. The HBM has been one of the most widely used theories in understanding health and illness behaviors, and due to its design, it has been previously used in vaccination studies to identify behavior relationships [35,36]. When compared with other models that explain behavior and resulting actions, the HBM was specifically developed to focus on preventative health research [35-38], which has been modified since its early use in the 1950s to be more inclusive and encourage interventions that improve health behaviors [39]. Thus, the HBM was chosen as the preferred model to investigate intention and behavior regarding COVID-19 vaccination. There are six main components of the HBM: perceived susceptibility, perceived severity, perceived benefits, perceived barriers, self-efficacy for health protective behaviors, and cues to action [40]. Previous studies, including those on H1N1 [41], hepatitis [42], human papillomavirus (HPV) [43], and measles [44], have identified HBM factors as important predictors of vaccination intentions. Therefore, it is necessary to explore the possible influence of these factors on people's willingness to vaccinate against COVID-19 in order to improve individual immunity and slow the epidemic. Although the aforementioned studies suggested that there were associations between HBM constructs and vaccine acceptance or hesitancy, relatively few studies have focused on COVID-19 vaccination behavior, especially in China and other countries where vaccinations are available to the domestic population [45].

In summary, we explored whether HBM constructs were associated with vaccine hesitancy and vaccination at the time when COVID-19 vaccination became available to the public in mainland China. A previous study identified that vaccine intention and willingness were important predictors of vaccination behavior, with more than 50% of the explained variance in influenza [46] and HPV [34] vaccinations. However, a gap seems to exist between intention and vaccination behavior [47], such as the willingness of students to receive the HPV vaccine predicting less than 10% of actual vaccinations [34]. Our first hypothesis (Hypothesis 1) was that vaccine hesitancy was negatively associated with COVID-19 vaccination behavior. In particular, we examined our major hypothesis (Hypothesis 2), which was that the HBM constructs of perceived barriers, self-efficacy, and cues to action would predict vaccine hesitancy and vaccination behavior. As in a previous study, self-efficacy is defined as the confidence in one’s ability to facilitate decisions to carry out a health behavior such as vaccination, which is useful only to the extent that one feels one can adequately implement the steps needed to perform the behavior [48]. Evidence based on the HBM poses several mechanisms regarding how self-efficacy is associated with vaccine intention and behaviors. Self-efficacy was able to mediate the relationship between perceived barriers to HPV vaccination and HPV vaccine intentions among young women [49]. A similar mediation effect was found in the association between perceived severity and susceptibility and the intent to receive the Zika vaccine [50]. It was also suggested that self-efficacy could influence the path from cues to action (eg, physician recommendation, family members recommendation, media coverage, and public health communication) to HPV vaccine uptake [51] and acceptance of the H1N1 vaccine [52]. The aforementioned studies suggested our third major hypothesis (Hypothesis 3), which was that self-efficacy of the COVID-19 vaccine would mediate the influence of other HBM constructs on vaccine hesitancy and vaccination.

## Methods

### Study Design and Participants

From January 1 to 24, 2021, we used convenience and snowball sampling to recruit a sample of 2580 participants from 31 out of a total of 34 provinces and autonomous regions in China, with each area consisting of at least 30 participants; we then conducted a web-based cross-sectional study. A digital questionnaire link was sent to a WeChat “Friends circle,” a function that can be used to share personal photos or public website links in one’s “Moments” to make them visible to friends on platforms such as Twitter and Facebook. This questionnaire link, on the Wenjuanxing platform, could then be forwarded or shared by participants with friends in their WeChat contact list whom they considered appropriate for this survey; their friends were also encouraged to send the link to their friend networks. The snowball sampling process continued until a sufficient sample size was reached. The first page of the questionnaire contained an electronic consent form. Each respondent received a small monetary reward of ¥5 (a currency exchange rate of ¥1=US $0.15 is applicable) after authentically completing the questionnaire, which took approximately 5 to 10 minutes. To prevent repeated entries from the same individual, who may attempt multiple entries for the enrollment reward, additional measures were adopted: (1) the same IP address was only allowed to be used once to fill in the questionnaire, which was a built-in function of the Wenjuanxing platform, and (2) participants were only allowed to fill in questionnaires after logging in to their WeChat accounts—they needed to register with this platform with a personal identity card—and each WeChat account could only be used once to fill in the questionnaire. The minimum sample size was calculated to be 1100 by using the following formula:




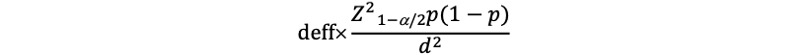




where the latest reported prevalence of COVID-19 vaccination hesitancy (p) was 35.5%, based on research that was conducted in China, nationwide, from January 10 to January 22, 2021 [53]. The type I error () was .05; thus, z_1-/2_=1.96, the precision (d) was 0.04, and the design effect (deff) was 2 [54]. The inclusion criteria for participants’ enrollment were as follows: (1) aged 18 to 59 years, (2) able to understand the questionnaire by themselves, and (3) could use online services, such as mobile phones, computers, and tablet computers. The questionnaires of participants who met the following exclusion criteria were discarded: (1) aged less than 18 years (n=16) or more than 59 years (n=32) and not eligible for vaccination until April 2021 in China and (2) returned invalid questionnaires (n=32). Questionnaires were deemed invalid if the following occurred: (1) participant gave one or two wrong answers to two quality control questions, including “Where is capital of China?” and “What’s three plus five?”; (2) occurrence of a logic check result error, which occurred when the participant selected both “no disease” and “any type of disease” in response to the question “Do you have any type of the following diseases or diagnosed medical histories”; and (3) participant took less than the minimum time of 3 minutes to complete the questionnaire. Cognitive interviewing with 5 subjects was done to refine the questionnaires through the web-based platform WeChat. Participants were required to respond to each item by answering three questions: (1) “What does ‘……’ mean to you?”, (2) “Can you repeat this question in your own words?”, and (3) “When you think about ‘……’ what comes to your mind?” We also asked participants to answer three questions for the overall survey, including the following: (1) “Are there additional questions you believe should be asked?”, (2) “Are there questions you believe should be deleted?”, and (3) “Are there questions you believe should be modified?” The entire questionnaire was tested and modified to appropriately conduct the survey. Finally, 2531 participants were included in this study. All participants consented to written ethics approval before the survey was conducted. This study was approved by the Institutional Review Board of Fudan University, School of Public Health (IRB00002408&FWA00002399), and approval expired on March 3, 2021.

### Measurements

#### Vaccine Hesitancy and Vaccination

Vaccine hesitancy was assessed with a one-item self-report measure that quantified the demand for, and acceptance of, vaccination: “How willing would you be to get the COVID-19 vaccine?” The respondents were asked to answer the question using the following 7-point scale recommended by the SAGE working group on vaccine hesitancy: “accept all [vaccines],” “accept but unsure,” “accept some,” “delay,” “refuse some,” “refuse but unsure,” and “refuse all” [17]. Vaccine hesitancy was defined as any response on the scale except for “accept all” or “accept but unsure.” Vaccination was assessed by asking the participants to answer “yes” or “no” to a single question: “Have you gotten the COVID-19 vaccine?”

#### Health Belief Model

Items derived from the HBM were adopted from a previous study or modified to measure the participants’ beliefs about COVID-19 vaccination. Five essential dimensions of health beliefs were measured as follows: (1) perceived susceptibility to COVID-19 in the future (three items; eg, “I was vulnerable to infection with SARS-CoV-2”), (2) perceived severity of COVID-19 infection (four items; eg, “It would be very harmful for me if I got COVID-19”), (3) perceived benefits of COVID-19 vaccination (three items; eg, “COVID-19 vaccination can protect me from infection with SARS-CoV-2”), (4) perceived barriers to COVID-19 vaccination (six items; eg, “The COVID-19 vaccine might have side effects, such as fever or soreness in the arm”), and (5) self-efficacy for COVID-19 vaccination (five items; eg, “I believe I can deal with side effects of the COVID-19 vaccine with doctors’ help”). Cues to action refer to external recommendations that might affect individuals’ health-related behaviors. In this study, the Cronbach α coefficients indicating internal consistency (ie, reliability) were .78 for the total HBM factors, .84 for perceived susceptibility to COVID-19, .80 for perceived severity of COVID-19 infection, .83 for perceived benefits of COVID-19 vaccination, .80 for perceived barriers to COVID-19 vaccination, and .82 for self-efficacy for COVID-19 vaccination. The sampling adequacy for the HBM factor scale was excellent (Kaiser-Meyer-Olkin=0.82). Inter-item correlations were sufficiently large for principal component analysis (PCA) (Bartlett test of sphericity: ^2^_210_=23,122.6, *P*<.001). The PCA revealed five factors, which in combination explained 68.58% of the variance, and each factor accounted for 24.23%, 20.55%, 10.32%, 8.16%, and 5.32% of the explained variance, respectively. An examination of the factor loadings after rotation suggested, as expected, that factor 1 (perceived barriers to COVID-19 vaccination) had six items with loading factors between 0.74 and 0.79, factor 2 (self-efficacy for COVID-19 vaccination) included five items with loading factors between 0.71 and 0.80, factor 3 (perceived severity of COVID-19 infection) included four items with loading factors between 0.67 and 0.85, factor 4 (perceived benefits of COVID-19 vaccination) included three items with loading factors between 0.68 and 0.85, and factor 5 (perceived susceptibility to COVID-19) included three items with loading factors between 0.78 and 0.89.

#### External Cues to Action

External cues to action were assessed based on four cues used in previous surveys [36,55]: recommendations from authorities, recommendations from friends or family, vaccination of authorities, and vaccination of friends or family. Participants were asked to state their level of agreement with each of the statements, with a score of 1 for positive responses (strongly agree or agree) and a score of 0 for neutral or negative responses (neither agree nor disagree, disagree, or strongly disagree). The Cronbach α coefficient for cues to action was .82. The sampling adequacy for the cues to action scale was excellent (Kaiser-Meyer-Olkin=0.75). Inter-item correlations were sufficiently large for PCA (Bartlett test of sphericity: ^2^_6_=2829.1, *P*<.001). The PCA revealed a single factor, which in combination explained 59.72% of the variance, and an examination of the factor loadings after rotation suggested, as expected, that the single factor included four items whose loading factors were between 0.65 and 0.84.

#### Demographic and Health-Related Characteristics

Demographic characteristics in this study included gender, age, educational level (high school degree and below, bachelor’s degree, or master’s degree and above), marital status (married or not married [including unmarried, divorced, and widowed]), occupation (medical worker or nonmedical worker), region (urban or rural), monthly salary (<¥6000, ¥6000-¥10,000, or >¥10,000), and family members with backgrounds in medical work or with medical education (yes or no). Health-related characteristics included self-rated health and self-reported chronic diseases having been diagnosed by doctors. Self-rated health was evaluated by a single question: “How is your perceived health in general?”; responses included “excellent,” “very good,” “good,” “general,” or “poor” [56]. We listed 16 common chronic diseases, such as hypertension and diabetes, and categorized the number of reported chronic diseases into 0, 1 or 2, and 3 or over.

#### Statistical Analysis

**Figure 1:**
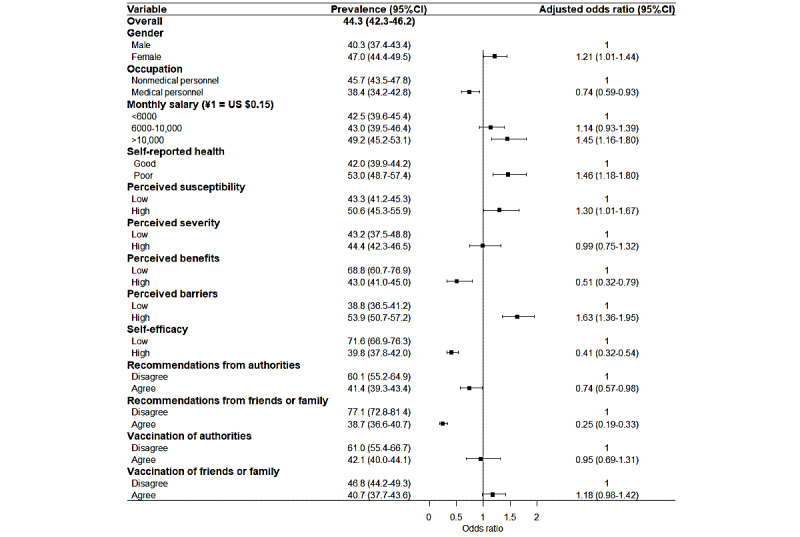
Associations between the health belief model and vaccine hesitancy.

**Figure 2:**
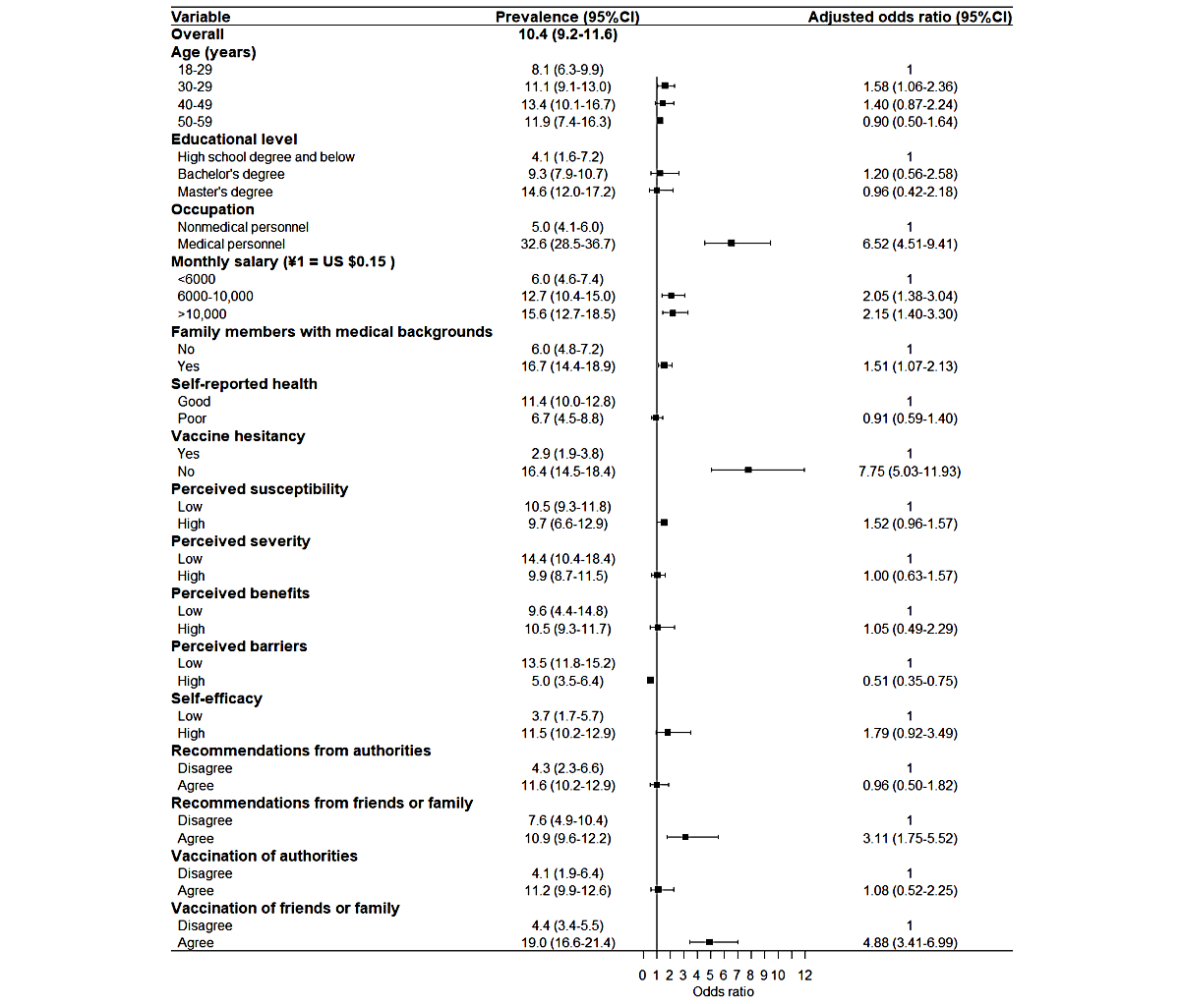
Associations between the health belief model and vaccination rate.

Frequencies were first calculated for all variables, and the prevalence and 95% CIs of vaccine hesitancy and vaccination were determined according to the participants’ demographics, health-related characteristics, and HBM factors. Multivariable logistic regression analyses were used to explore the demographic and health-related characteristics (Table 1) as well as the HBM factors (Table 2) associated with vaccine hesitancy and vaccination. We then ran the multivariable logistic regression again to determine the HBM factors associated with vaccine hesitancy and vaccination after controlling for covariates (ie, demographic and health-related characteristics), with a significance level of *P*<.05. Odds ratios (ORs) with 95% CIs were calculated for each independent variable and were visualized in forest plots (Figures 1 and 2). All of the analyses were performed using SAS software, version 9.4 (SAS Institute Inc), and all tests were two-tailed with a significance level of *P*<.05. We used the forest plot package in R software, version 3.5.3 (The R Foundation), to generate the forest plots. We used Mplus, version 8.4 (Muthén & Muthén), to establish structural equation modeling (SEM) and to assess the standardized structure coefficients (SSCs) among the HBM factors of vaccine hesitancy and vaccination. The mean- and variance-adjusted weighted least squares method was employed as the method of estimation because the analyses included categorical endogenous variables (ie, vaccine hesitancy and vaccination), and the link was the probability unit in the current model [57]. We freed covariances between error terms based on their modification indices during the estimation process to improve model fit. The most common indices and acceptable reference values included the magnitude of χ^2^ divided by its degrees of freedom (χ^2^/df <5), the comparative fit index (CFI >0.90), the Tucker-Lewis index (TLI >0.90), and the root mean square error of approximation (RMSEA <0.08), which were used to determine whether the data fit the model [58].

## Results

### Participant Characteristics

Our analysis included 2531 participants aged between 18 and 59 years (mean 33.92 years, SD 8.94); 58.7% (1486/2531) of the participants were female. Most of the participants were married (1660/2531, 65.6%), had a bachelor’s degree (1609/2531, 63.6%), were nonmedical personnel (2034/2531, 80.4%), lived in urban areas (2262/2531, 89.4%), reported good health (2020/2531, 79.8%), and did not have chronic diseases (1617/2531, 63.9%). Slightly less than half of the participants reported monthly salaries lower than ¥6000 (1128/2531, 44.6%) and had family members with medical personnel backgrounds (1056/2531, 41.7%) (Table 1).

**Table 1:** Distribution of vaccine hesitancy and vaccination rate by participant demographics and health-related characteristics.

Characteristics		Participants (N=2531), n (%)	Vaccine hesitancy				Vaccination rate		
			Vaccine hesitancy, n (%)	OR^a^ (95% CI)	*P* value	Vaccination, n (%)		OR (95% CI)	*P* value
**Age (years)**									
	18-29	926 (36.6)	412 (44.5)	1		75 (8.1)		1	
	30-39	993 (39.2)	467 (47.0)	1.11 (0.93-1.33)	.27	110 (11.1)		1.41 (1.04-1.92)	.03
	40-49	410 (16.2)	163 (39.8)	0.82 (0.65-1.04)	.11	55 (13.4)		1.76 (1.22-2.54)	.003
	50-59	202 (8.0)	78 (39.6)	0.79 (0.58-1.07)	.13	24 (11.9)		1.53 (0.94-2.49)	.09
**Gender**									
	Male	1045 (41.3)	422 (40.4)	1		116 (11.1)		1	
	Female	1486 (58.7)	698 (47.0)	1.31 (1.11-1.53)	.001	148 (10.0)		0.89 (0.69-1.15)	.36
**Marital status**									
	Married	1660 (65.6)	725 (43.7)	1		187 (11.3)		1	
	Not married	871 (34.4)	395 (45.4)	1.07 (0.91-1.26)	.42	77 (8.8)		0.76 (0.58-1.01)	.06
**Educational level**									
	High school degree and below	204 (8.0)	83 (40.7)	1		9 (4.4)		1	
	Bachelor’s degree	1609 (63.6)	725 (45.1)	1.20 (0.89-1.61)	.24	150 (9.3)		2.23 (1.12-4.43)	.02
	Master’s degree and above	718 (28.4)	312 (43.5)	1.12 (0.82-1.54)	.48	105 (14.6)		3.71 (1.84-7.47)	<.001
**Occupation**									
	Nonmedical personnel	2034 (80.4)	929 (45.7)	1		102 (5.0)		1	
	Medical personnel	497 (19.6)	191 (38.4)	0.74 (0.61-0.91)	.004	162 (32.6)		9.16 (6.97-12.04)	<.001
**Region**									
	Urban	2262 (89.4)	1016 (44.9)	1		242 (10.7)		1	
	Rural	269 (10.6)	104 (38.7)	0.77 (0.60-1.00)	.05	22 (8.2)		0.74 (0.47-1.17)	.20
**Monthly salary (¥)^b^**									
	<6000	1128 (44.6)	478 (42.5)	1		68 (6.0)		1	
	6000-10,000	787 (31.1)	338 (43.0)	1.02 (0.85-1.23)	.83	100 (12.7)		2.27 (1.64-3.13)	<.001
	>10,000	616 (24.3)	303 (49.2)	1.31 (1.08-1.60)	.007	96 (15.6)		2.88 (2.07-3.99)	<.001
**Family members with medical backgrounds**									
	No	1475 (58.3)	667 (45.2)	1		88 (6.0)		1	
	Yes	1056 (41.7)	453 (42.9)	0.91 (0.78-1.06)	.25	176 (16.7)		3.13 (2.28-4.17)	<.001
**Self-reported health**									
	Good	2020 (79.8)	849 (42.0)	1		230 (11.4)		1	
	Poor	511 (20.2)	271 (53.0)	1.56 (1.28-1.89)	<.001	34 (6.7)		0.55 (0.38-0.81)	.002
**Number of chronic diseases**									
	0	1617 (63.9)	688 (42.3)	1		178 (11.0)		1	
	1	639 (25.4)	300 (47.0)	0.84 (0.70-1.01)	.045	64 (10.0)		1.11 (0.82-1.50)	.49
	2 and above	288 (11.2)	136 (49.5)	1.13 (0.86-1.49)	.49	22 (8.0)		0.78 (0.47-1.30)	.34
**Vaccine hesitancy**									
	Yes	1120 (44.3)	N/A^c^	N/A	N/A	32 (2.9)		1	
	No	1411 (55.7)	N/A	N/A	N/A	232 (16.4)		6.69 (4.58-9.77)	<.001

^a^OR: odds ratio.

^b^A currency exchange rate of ¥1=US $0.15 is applicable.

^c^N/A: not applicable.

### Distribution of Vaccine Hesitancy and Vaccination by Participant Characteristics and Health Belief Model Factors

Overall, 44.3% (1120/2531; 95% CI 42.3%-46.2%) of the participants were classified as vaccine hesitant: 1.4% responded “refuse all,” 5.3% responded “refuse but unsure,” 3.7% responded “refuse some,” 18.8% responded “delay,” and 15.1% responded “accept some.” Overall, 55.7% (1411/2531) of the participants were classified as vaccine accepting: 25.1% responded “accept but unsure” and 30.6% responded “accept all.” Only 10.4% (264/2531; 95% CI 9.2%-11.6%) of the participants had been vaccinated for COVID-19, while the majority (2267/2531, 89.6%) had not been.

According to the multivariable logistic regression analyses including participant characteristics (Table 1), the participants were more likely to be vaccine hesitant if they were female (OR 1.31, 95% CI 1.11-1.53), were nonmedical personnel (OR 1.35, 95% CI 1.10-1.64), had poor self-rated health (OR 1.56, 95% CI 1.28-1.89), or had a monthly salary over ¥10,000 (OR 1.31, 95% CI 1.08-1.60). The participants were more likely to have been vaccinated if they were 30 to 39 years old (OR 1.41, 95% CI 1.04-1.92), had a bachelor’s degree (OR 2.23, 95% CI 1.12-4.43), had a master’s degree and above (OR 3.71, 95% CI 1.12-4.43), were medical personnel (OR 3.71, 95% CI 1.12-4.43), had good self-rated health (OR 1.82, 95% CI 1.23-2.63), were not vaccine hesitant (OR 6.69, 95% CI 4.58-9.77), had a monthly salary between ¥6000 and ¥10,000 (OR 2.27, 95% CI 1.64-3.13), had a monthly salary over ¥10,000 (OR 2.88, 95% CI 2.07-4.17), or had family members with medical personnel backgrounds (OR 3.13, 95% CI 2.28-4.17).

According to the multivariable regression analyses including the HBM factors (Table 2), the participants were more likely to be vaccine hesitant if they had high perceived susceptibility to COVID-19 (OR 1.34, 95% CI 1.07-1.69) or had high perceived barriers to vaccination (OR 1.84, 95% CI 1.56-2.17). The participants were less likely to be vaccine hesitant if they had high perceived benefits of vaccination (OR 0.34, 95% CI 0.23-0.50), had high self-efficacy for vaccination (OR 0.26, 95% CI 0.20-0.34), agreed with recommendations from authorities (OR 0.47, 95% CI 0.38-0.58), agreed with recommendations from friends or family (OR 0.19, 95% CI 0.14-0.24), agreed with the vaccination of authorities (OR 0.46, 95% CI 0.36-0.60), or agreed with the vaccination of friends or family (OR 0.77, 95% CI 0.66-0.91). The participants were more likely to have been vaccinated if they had high self-efficacy for vaccination (OR 3.39, 95% CI 1.92-6.00), agreed with recommendations from authorities (OR 2.89, 95% CI 1.75-4.78), agreed with the vaccination of authorities (OR 2.94, 95% CI 1.62-5.31), or agreed with the vaccination of friends or family (OR 5.05, 95% CI 3.77-6.76).

**Table 2:** Distribution of vaccine hesitancy and vaccination by health belief model (HBM) factors and cues to action.

HBM factors and cues to action			Participants (N=2531), n (%)		Vaccine hesitancy							Vaccination				
					Vaccine hesitancy, n (%)		OR^a^ (95% CI)		*P* value		Vaccination, n (%)			OR (95% CI)		*P* value
**Perceived susceptibility**																
	Low	2191 (86.6)		948 (43.3)		1				231 (10.4)			1			
	High	340 (13.4)		172 (50.6)		1.34 (1.07-1.69)		.01		33 (9.7)			0.91 (0.62-1.34)		.64	
**Perceived severity**																
	Low	292 (11.5)		126 (43.2)		1				42 (14.4)			1			
	High	2239 (88.5)		994 (44.4)		1.05 (0.82-1.36)		.69		222 (9.9)			0.66 (0.46-0.93)		.02	
**Perceived benefits**																
	Low	125 (4.9)		86 (68.8)		1				12 (9.6)			1			
	High	2406 (95.1)		1034 (43.0)		0.34 (0.23-0.50)		<.001		252 (10.5)			1.01 (0.95-1.07)		.76	
**Perceived barriers**																
	Low	1622 (64.1)		630 (38.8)		1				219 (13.5)			1			
	High	909 (35.9)		490 (53.9)		2.08 (1.77-2.45)		<.001		49 (5.0)			0.33 (0.24-0.47)		<.001	
**Self-efficacy**																
	Low	352 (13.9)		252 (71.6)		1				13 (3.7)			1			
	High	2179 (86.1)		868 (39.8)		0.26 (0.20-0.34)		<.001		251 (11.5)			3.39 (1.92-6.00)		<.001	
**Recommendations from authorities**																
	Disagree	393 (15.5)		236 (60.1)		1				17 (4.3)			1			
	Agree	2138 (84.5)		884 (41.4)		0.47 (0.38-0.58)		<.001		247 (11.6)			2.89 (1.75-4.78)		<.001	
**Recommendations from friends or family**																
	Disagree	367 (14.5)		283 (77.1)		1				28 (7.6)			1			
	Agree	2164 (85.5)		837 (38.7)		0.19 (0.14-0.24)		<.001		236 (10.9)			1.48 (0.99-2.23)		.06	
**Vaccination of authorities**																
	Disagree	290 (11.5)		177 (61.0)		1				12 (4.1)			1			
	Agree	2241 (88.5)		943 (42.1)		0.46 (0.36-0.60)		<.001		252 (11.2)			2.94 (1.62-5.31)		<.001	
**Vaccination of friends or family**																
	Disagree	1488 (58.8)		696 (46.8)		1				66 (4.4)			1			
	Agree	1043 (41.2)		424 (40.7)		0.77 (0.66-0.91)		.002		198 (19.0)			5.05 (3.77-6.76)		<.001	

^a^OR: odds ratio.

### Influencing Factors of Vaccine Hesitancy and Vaccination

We included the participant characteristics and HBM factors in the vaccine hesitancy logistic regression, and the influencing factors are shown in Figure 1. The risk factors for vaccine hesitancy were female gender (*P*=47.0%, 95% CI 44.4%-49.5%; OR 1.12, 95% CI 1.01-1.44), monthly salary over ¥10,000 (*P*=49.2%, 95% CI 45.2%-53.1%; OR 1.45, 95% CI 1.16-1.80), poor self-rated health (*P*=53.0%, 95% CI 48.7%-57.4%; OR 1.46, 95% CI 1.18-1.80), high perceived susceptibility to COVID-19 (*P*=50.6%, 95% CI 45.3%-55.9%; OR 1.30, 95% CI 1.01-1.67), and high perceived barriers to vaccination (*P*=53.9%, 95% CI 50.7%-57.2%; OR 1.63, 95% CI 1.36-1.95). Additionally, the protective factors against vaccine hesitancy were occupation as medical personnel (*P*=38.4%, 95% CI 34.2%-42.7%; OR 0.74, 95% CI 0.59-0.93), high perceived benefits of vaccination (*P*=43.0%, 95% CI 41.0%-45.0%; OR 0.51, 95% CI 0.32-0.79), high self-efficacy for vaccination (*P*=38.4%, 95% CI 48.7%-57.4%; OR 1.46, 95% CI 1.18-1.80), agreement with recommendations from authorities (*P*=41.4%, 95% CI 39.3%-43.4%; OR 0.74, 95% CI 0.57-0.98), and agreement with recommendations from friends or family (*P*=41.4%, 95% CI 39.3%-43.4%; OR 0.74, 95% CI 0.57-0.98).

We included the participant characteristics, the HBM factors, and vaccine hesitancy in the vaccination logistic regression, and the influencing factors are shown in Figure 2. The promoting factors for vaccination were occupation as medical personnel (*P*=32.6%, 95% CI 28.5%-36.7%; OR 6.52, 95% CI 4.51-9.41), monthly salary between ¥6000 and ¥10,000 (*P*=12.7%, 95% CI 10.4%-15.0%; OR 2.05, 95% CI 1.38-3.04), monthly salary over ¥10,000 (*P*=15.6%, 95% CI 12.7%-18.5%; OR 2.15, 95% CI 1.40-3.30), family members with medical personnel backgrounds (*P*=16.7%, 95% CI 14.4%-18.9%; OR 1.51, 95% CI 1.07-2.13), a lack of vaccine hesitancy (*P*=16.4%, 95% CI 14.5%-18.4%; OR 7.75, 95% CI 1.01-1.67), agreement with recommendations from friends or family (*P*=10.9%, 95% CI 9.6%-12.2%; OR 3.11, 95% CI 1.75-5.52), and agreement with the vaccination of friends or family (*P*=19.0%, 95% CI 19.6%-21.4%; OR 4.88, 95% CI 3.41-6.99). Additionally, a lower vaccination rate was associated with higher perceived barriers to COVID-19 vaccination (p=5.0%, 95% CI 3.5%-6.4%; OR 0.51, 95% CI 0.35-0.75).

### Structural Equation Modeling of Vaccination

**Figure 3:**
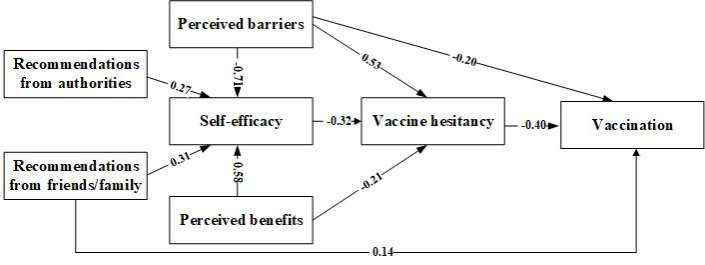
The paths among vaccine hesitancy, vaccination, and health belief model factors. The numbers on the lines are the standardized structure coefficients.

We used SEM to examine the underlying psychological mechanism of vaccination behavior (Figure 3). Based on the goodness-of-fit statistics, SEM showed a better fit to the data than the regression models (χ^2^/df=4.62; RMSEA=0.05; CFI = 0.95; TLI = 0.91), and all of the paths were statistically significant (*P*<.05). The findings suggested that a mediating effect of self-efficacy, influenced by perceived barriers (SSC=−0.71, *P*<.001), perceived benefits (SSC=0.58, *P*<.001), agreement with recommendations from authorities (SSC=0.27, *P*<.001), and agreement with recommendations from friends or family (SSC=0.31, *P*<.001), was negatively associated with vaccination (SSC=−0.45, *P*<.001) via vaccine hesitancy (SSC=−0.32, *P*<.001). Additionally, perceived barriers (SSC=0.53, *P*<.001) and perceived benefits (SSC=−0.21, *P*<.001) were directly associated with vaccine hesitancy. Perceived barriers (SSC=−0.20, *P*<.001) and recommendations from friends or family (SSC=0.14, *P*<.001) were directly correlated with vaccination behavior.

### Availability of Data and Materials

The data that support the findings of this study are available from the School of Public Health, Fudan University. The data were used under license for this study and are not publicly available. The data are, however, available from the authors upon reasonable request and with permission from the School of Public Health, Fudan University.

## Discussion

### Principal Findings

The findings of our study suggest that five HBM constructs—in the absence of perceived barriers, a high level of perceived benefits, and self-efficacy—as well as individuals’ agreement with recommendations from authorities and friends or family were negatively associated with COVID-19 vaccine hesitancy and positively associated with vaccination behavior. Furthermore, psychological mechanisms were found to mediate the relationship between perceived barriers, perceived benefits, recommendations from authorities and friends or family, and vaccination uptake behavior via vaccine hesitancy. Self-efficacy, perceived barriers, and perceived benefits were correlated with vaccine hesitancy, while perceived barriers and recommendations from friends or family were directly correlated with vaccination.

In this study based in China, the prevalence of vaccine hesitancy was 44.3% (95% CI 42.3%-46.2%), and the vaccination rate was 10.4% (95% CI 9.2%-11.6%), representing high vaccine hesitancy and low vaccination behaviors. Vaccine hesitancy has been universally reported in recent research, with over half of participants (53%) across 19 countries showing vaccine hesitancy, which is similar to our results [59] and in accordance with the decline in vaccine acceptance (from >70% in March 2020 to <50% in October 2020) reported by a recent review [60]. Undoubtedly, eliminating vaccine hesitancy would be beneficial to voluntary vaccination behaviors, as seen in this study, which showed that the vaccination rate was nearly 8 times higher among the participants who were accepting of vaccines compared to those who were vaccine hesitant. In the SEM results, vaccine hesitancy was also strongly negatively associated with vaccination behaviors (Hypothesis 1 confirmed). Therefore, the control of vaccine hesitancy and the promotion of voluntary vaccination still seem to be challenges in the context of the COVID-19 pandemic.

In this study, female participants showed more COVID-19 vaccine hesitancy, which is consistent with previous findings in the literature [61,62]; a possible reason for this finding is that women are more likely to be concerned about side effects [63] and take nonpharmaceutical protective measures (eg, masking and maintaining social distance) [64], while men are more inclined to adopt medical intervention [65]. Medical personnel showed less vaccine hesitancy and a much higher vaccination rate in this study, which may be inconsistent with the general argument that health workers have strong negative attitudes toward vaccines, with strong skepticism about their safety and effectiveness, especially regarding the influenza vaccine [66,67]. Another finding seems unexpected; that is, that the participants with higher monthly salaries were associated with both vaccine hesitancy and a higher vaccination rate; in other words, even though these individuals were vaccine hesitant, they were still vaccinated. Vaccine hesitancy was not only a direct determinant of vaccination but also a perceived barrier. Participants with higher salaries were more likely to have higher socioeconomic status [68], so they could more easily access social resources; that is, they had lower barriers to obtaining vaccines, which could then increase the vaccination rate among this group.

Although some of the HBM factors were not directly associated with the vaccination rate, perceived benefits of vaccination, perceived barriers to vaccination, self-efficacy for vaccination, and recommendations from authorities were correlated with vaccine hesitancy (Hypothesis 2 partially confirmed), which was consistent with previous research among the Malaysian public [36] and the Chinese general population [69]. In all HBM constructs associated with vaccine hesitancy and vaccination, self-efficacy for COVID-19 vaccination was an important predictor of vaccination behaviors, via vaccine hesitancy. This result is similar to the findings of previous studies on influenza vaccination, according to which self-efficacy is a key factor of willingness, which in turn predicts behavior [46,70]. Self-efficacy also plays a mediating role between vaccine hesitancy and other HBM components, including perceived barriers, perceived benefits, and recommendations from authorities and friends or family, and it indirectly influences vaccination uptake. This finding was supported by the HBM hypothesis (Hypothesis 3 partially confirmed) that HBM constructs and cues to action may not share a juxtaposition or parallel relationship, but self-efficacy functioned as a serial mediator [71]. Hilyard et al noted that public self-efficacy for COVID-19 vaccination could be promoted by enhancing the perceived benefits of vaccination, confidence in overcoming possible side effects (ie, perceived barriers), and recommendations from authorities, such as the Obamas’ modeling of H1N1 vaccine acceptance for their daughters [52]. In this study, self-efficacy was measured as a specific domain with confidence in the safety of the COVID-19 vaccine, a low prevalence of side effects of the COVID-19 vaccine, and success in dealing with side effects. Vaccine safety or side effects, which are regarded as contributing to the development of disease, are of paramount importance to individual efficacy when deciding whether to vaccinate [72,73] and are even relevant aspects that help explain the antivaccine movement in Europe [74]. A study argues that a perceived risk-benefit balance may influence confidence in vaccine uptake; in other words, a combined decision-making process relies on a trade-off between benefits and risks [66]. In addition to cues to action, this result was consistent with a previous study showing that compliance with recommendations from health workers may also be correlated with confidence in vaccine efficacy [73], because they can share personal knowledge about being immunized and motivate vaccine uptake efficacy [75].

In addition to the direct and mediating effect of self-efficacy, some HBM constructs were directly associated with vaccine hesitancy and vaccination behavior. Perceived barriers were both positively correlated with vaccine hesitancy and detrimental to vaccination, as measured by the safety, side effects, and inaccessibility of the COVID-19 vaccine, in which safety may influence self-efficacy as aforementioned, while inaccessibility would hinder the perceived convenience of COVID-19 vaccination behavior directly. With a more specific formulation, a controlled before-and-after trial study showed that arranging time and transportation were key predictors of both intention and behavior regarding influenza vaccination [76]. A previous survey also found that the side effects and safety of influenza vaccination were the most common reasons for vaccine hesitancy [77]. Perceived benefits were associated with vaccine hesitancy, which was measured by preventing the self and one’s family from being infected after COVID-19 vaccination. From an altruistic motivation perspective, people could be vaccinated to protect not only themselves but also their loved ones; in other words, there could be more willingness to receive the vaccine if individuals believe that it helps reduce the transmission of COVID-19 [78]. Recommendations from family were found to be directly associated with vaccination behavior in this study. An online survey in Canada showed that respondents reported that encouragement from both colleagues and employers was beneficial to their vaccination decision-making process [55]. Another finding implied that a recommendation from a spouse or a friend is an important cue to action in determining willingness to accept the Zika virus vaccine [79]. However, perceived susceptibility and severity were not enough to reduce vaccine hesitancy and promote vaccination behavior. A review indicated that perceived barriers were the most powerful single predictor of preventive health behavior across all studies and behaviors, and perceived severity was the least powerful predictor [71].

From the perspective of the HBM on understanding vaccination behavior, it is valuable that self-efficacy is an important and direct predictor of COVID-19 vaccine hesitancy because it can also mediate the influences from cues to action, perceived barriers, and perceived benefits. Furthermore, vaccine hesitancy was strongly correlated with vaccination behavior but was not the only determinant, since perceived barriers and recommendations from friends or family were also associated with vaccination behavior directly and in combination.

In practice, it is valuable for other nations to know what the Chinese vaccine hesitancy and vaccination statuses were at the beginning of the critical period when COVID-19 vaccination became available to the public, free of charge. This finding indicates that health authorities or doctors may be less effective in motivating people to action, while it may be useful to advocate for more volunteers to engage in motivating their friends or family members. Although the antivaccine movement that occurred in other nations was not popular in mainland China, vaccine hesitancy and refusal were not rare occurrences without mandatory vaccination in this study. Moreover, it is essential to reinforce the publishing of information regarding the safety and validity of COVID-19 vaccines and incentives of vaccination completion, which could then promote public confidence in overcoming vaccination barriers and in achieving benefits after vaccination.

In summary, there was a high prevalence of vaccine hesitancy and low vaccination behavior in China during the first month (January 2021) when vaccinations became available to the vaccine-eligible population. The HBM framework is a useful framework to guide the development of future campaigns to reduce vaccine hesitancy and promote COVID-19 vaccination.

### Limitations

There are some potential limitations to this study. First, due to the convenience sampling and snowball recruitment methods that were part of the online survey process, selection bias, such as the participation of fewer respondents with low education attainment and fewer older adults (aged over 50 years), may have affected the generalizability of the results. Second, vaccine hesitancy was measured by a single item derived from a definition from the SAGE working group, which may promote more accurate measurement tools in future research. Furthermore, the vaccination rate in this study may not reflect future trends because only some participants had received the vaccine in a timely manner, vaccinations were available to the public for only 1 month, and there were no incentives except to receive a free vaccination before participating in the study.

## Abbreviations

CFI: comparative fit index
d: precision
deff: design effect
DIT: diffusion of innovation theory
HBM: health belief model
HPV: human papillomavirus
OR: odds ratio
p: prevalence of COVID-19 vaccination hesitancy
PCA: principal component analysis
RMSEA: root mean square error of approximation
SAGE: Strategic Advisory Group of Experts
SEM: structural equation modeling
SSC: standardized structure coefficient
TLI: Tucker-Lewis index
TPB: theory of planned behavior
WHO: World Health Organization
